# Underground diversity: Uropodina mites (Acari: Mesostigmata) from European badger (*Meles meles*) nests

**DOI:** 10.1007/s10493-020-00563-6

**Published:** 2020-10-24

**Authors:** Przemysław Kurek, Krzysztof Nowakowski, Tomasz Rutkowski, Agnieszka Ważna, Jan Cichocki, Michał Zacharyasiewicz, Jerzy Błoszyk

**Affiliations:** 1 Department of Plant Ecology and Environmental Protection, Adam Mickiewicz University, Uniwersytetu Poznańskiego 6, 61-614 Poznań, Poland; 2 Department of Zoology, Institute of Biological Sciences, University of Zielona Góra, Prof. Z. Szafrana 1, 65-516 Zielona Góra, Poland; 3 Natural History Collections, Adam Mickiewicz University, Uniwersytetu Poznańskiego 6, 61-614 Poznań, Poland; 4 Department of General Zoology, Adam Mickiewicz University, Uniwersytetu Poznańskiego 6, 61-614 Poznań, Poland

**Keywords:** Uropodina, Mites, *Meles meles*, Badger nests, Species diversity, *Nenteria oudemansi*, *Trematura patavina*

## Abstract

Badgers can gather huge quantities of organic material to build their nests for winter time and to rear their cubs. Moreover, badger burrows (setts) are characterized by specific microclimate with quite stable temperature and humidity. Their fauna is poorly studied, especially in respect of saprobiontic Uropodina mites. In 2018–2019, we monitored 94 badger setts to search for nest material that had been thrown away during cleaning of the chambers after mating and winter sleep. In the collected material from 32 badger nests, we found 413 Uropodina mites of 16 species, in various stages of development (adults, protonymphs, and deutonymphs). The community was dominated by three mite species: *Trematura patavina* (22.5%, *n* = 93), *Oodinychus ovalis* (17.2%, *n* = 71), and *Olodiscus minima* (15.5%, *n* = 64). Other nidicolous—i.e., nest-dwelling—species included: *Nenteria oudemansi* (14.8%, *n* = 61), *Phaulodiaspis borealis* (7.0%, *n* = 29), *Phaulodiaspis rackei* (4.6%, *n* = 19), *Uroseius hunzikeri* (1.7%, *n* = 7), *Uropoda orbicularis* (1.5%, *n* = 6), and *Apionoseius infirmus* (1.0%, *n* = 4). The most frequent species were: *Oodinychus ovalis* (62.5%, 20 nests), *N. oudemansi* (46.9%, 15 nests), and *Olodiscus minima* (40.6%, 13 nests). Detrended correspondence analysis indicated that the Uropodina community from badger nests differed from that of mole nests, studied earlier. In setts, the Uropodina community included *T. patavina* and *N. oudemansi*, which were for the first time recorded from underground badger nests. This is the first record of *N. oudemansi* from Poland.

## Introduction

European badger (*Meles meles*) burrows, also known as setts, play an important role in shaping species diversity in many habitats across Europe, e.g., in respect of vascular plants (Kurek et al. 2014), mosses (Kurek and Cykowska-Marzencka 2016), soil-dwelling animals (Rola et al. 2017), and fungi (Sleeman et al. 1995, 1997). This is caused by permanent digging out the soil from deeper horizons and creation of bare ground heaps near the numerous entrances to setts (Kurek 2019), which form elaborate systems of chambers connected by underground corridors (Roper 1992). Extensive setts result also in another, underground side of biodiversity shaped by badgers. Corridors and chambers may act as convenient shelters for many animals (e.g., Hancox 1988; Nowakowski et al. 2020), as setts are characterized by specific microclimate with quite stable temperature and humidity (Moore and Roper 2003; Kaneko et al. 2010). Moreover, badgers may gather up to 11 kg of organic material in chambers, where they spend winter time and rear the cubs (Roper 1992). Fischer and Dunand (2016) reported that the nest material may exceed the volume of 37 dm^3^. Such an accumulation of organic matter may create ideal conditions for many saprotrophic invertebrates, including mites (Acari). However, badgers are known to be hosts for only few mite species: *Demodex melesinus* (Izdebska et al. 2018) and *Sarcoptes scabiei* (Kołodziej-Sobocińska et al. 2014), both living in badger skin, and *Baloghella melis*, which is a saprophagous nest-inhabitant (Wurst and Pfister 1990). Despite the huge quantity of gathered organic material, the fauna inhabiting nests in badger burrows is still poorly studied, especially in respect of mites.

Earlier studies concerning the nidicolous (i.e., nest-dwelling) fauna of mammal burrows and warrens were mostly focused on small mammals dwelling in underground nests (Hackman 1963; Bartkowska 1986; Mašán and Stanko 2005; Mąkol et al. 2010; Kaminskienė et al. 2020). The nest fauna is usually represented by species whose reproductive cycles are closely connected with the burrow host, so it may be host-specific, numerously represented by ectoparasites (Howell 1960; Hancox 1980, 1988; Cox et al. 1999) or saprotrophic taxa connected with organic material hoarded in chambers (Seastedt et al. 1986). This applies especially to Uropodina mites, as a model group. They occur at all latitudes except polar regions, wherever any organic matter is accumulated (Napierała et al. 2016), and many of them live in very specific microhabitats, e.g., mammal nests (Błoszyk 1985; Błoszyk et al. 2003; Krawczyk et al. 2015). Such nidicolous Uropodina mites were considered in greatest detail with respect to moles, as a case study (Napierała and Błoszyk 2013). European mole *Talpa europaea* is a common species that gathers lots of diverse plant material in underground nests, in comparison to other small mammals, like rodents (Nowosad 1990; Napierała et al. 2016). Thus, mole is a good reference model to compare with mite assemblages from badger nests.

Reports presenting some mites from burrows of medium-sized carnivores (Howell 1960; Hancox 1988) allowed us to suspect that badger nests may be crucial places also for many Uropodina mite species dwelling in organic material stored underground. Moreover, badgers build nests in well-developed systems of setts, providing a microclimate with stable humidity (Moore and Roper 2003; Kaneko et al. 2010). This is a relevant factor for mites from the suborder Uropodina because mesohygrophilic species constitute the majority of this taxonomic group (Napierała and Błoszyk 2013). Thus, we supposed that badger burrows are convenient places for diverse mite fauna dwelling in nest material. Because of the lack of documented information about mite assemblages from badger nests, we aimed to analyse them quantitatively and qualitatively, in comparison to some data from similar microhabitats, such as mole nests. Because of the large size and specific environment of badger burrows, we hypothesized that they differ from mole burrows in respect of Uropodina communities.

## Methods

### Study area

Field research was carried out in a lowland region of western Poland near Trzciel (52°17′–52°32′ N, 15°30′–16°01′ E). The study area covered 389.5 km^2^, mostly a mosaic of forests and fields. Forests, forming 213 patches varying in size from 1 ha to more than 2000 ha, occupy 52% of the study area. Scots pine (*Pinus sylvestris*) on sandy soils is the dominant species. Four small rivers flow through the study area, and lakes cover about 1350 ha. The mild climate prevails, with an average annual temperature of 10.5 °C. The coldest month is February, with a mean temperature of −4.1 °C, and the warmest one is August, with a mean of 21.7 °C (meteomodel.pl).

### Sampling methods

In 2018–2019 we monitored 94 badger setts, including those used as breeding sites, to collect nest material that has been thrown away during cleaning the chambers, i.e., after mating and winter sleep. The setts were controlled in the autumn of 2018 (17 nests found) as well as spring of 2019 (7 nests) and autumn of 2019 (12 nests), when badgers started to be active after winter (February–March) and when they began preparing for the coming winter (ca. late September). Only fresh, moist nest material was sampled. In 10 cases, nest material was sampled in the same sett: in three setts we found nest material during three surveys (3 × 3 = 9 nests) and in seven setts during two surveys (7 × 2 = 14 nests). In the other 13 setts, nests were sampled only once, so the total number of found nests was 36. However, to avoid data duplication in dominance and frequency computations, we excluded four nests sampled in the autumn of 2019 from analyses, as they were obtained from the same setts as four nests sampled in the spring of the same season. Consequently, the final number of nests analysed in this study was 32.

Badgers throw away the nest material from the sett and leave it near the entrance—on top of the soil heaps or just below. The volume of nest material differed between setts and its dry mass varied (0.2–2.9 kg). Nest material consisted mostly of grass and Scots pine needles, and partially it was mixed with soil excavated from the burrow. All the organic material was sampled and transported to the laboratory. The mites were extracted with Tullgren funnels for 6 days, and preserved in 75% alcohol. Both permanent and temporary microscope slide preparations were made (using Hoyer’s medium), and the specimens were identified with the keys of (Kadite and Petrova 1977; Evans and Till 1979; Karg 1989; Błoszyk 1999; Mašán 2001). The samples were deposited in a soil-fauna collection (Natural History Collections, Faculty of Biology, Adam Mickiewicz University, Poznań).

### Data analysis

In accordance with Błoszyk (1999), the analysis of the Uropodina community was based on the indices of dominance (the number of individuals of *i*th species compared to individuals of all species in all samples) and frequency (the number of samples with *i*th species compared to all samples). The following classes of dominance were used: D5 = eudominants (> 30%); D4 = dominants (15.1–30.0%); D3 = subdominants (7.1–15.0%); D2 = residents (3.1–7.0%); and D1 = subresidents (≤3%). Frequency classes were: F5 = euconstants (> 50%); F4 = constants (30.1–50.0%); F3 = subconstants (15.1–30.0%); F2 = accessory species (5.1–15.0%); and F1 = accidentals (≤5%). The dominance and frequency characteristics were computed with data pooled for both seasons.

To assess the significance of differences in numbers of Uropodina species and individuals in badger nests between years, Student’s *t* test was applied with R v.3.5.3 software (R Core Team 2019). To obtain a normal or at least symmetric distribution of data, they were transformed with a logarithmic or exponential function. However, extensive analysis of seasonal changes in mite communities was not the aim of this paper. For Uropodina community analysis, the data from both seasons were pooled and subjected to detrended correspondence analysis (DCA, gradient length = 6.61) performed with CANOCO v.5 software (Šmilauer and Lepš 2014). To investigate relationships between badger nests and nests of other mammals that collect plant material in underground chambers, we used earlier published data from nests of moles (Błoszyk 1985). Numbers of mite individuals from mole and badger nests were standardized for DCA and expressed as proportions of given mite species to all species per sample. Uropodina specimens not determined to species level (one taxon) were treated as unverified records and excluded from further DCA analysis. This approach eliminated the influence of the unidentified species (*Uropoda* sp.) and improved the relation between the number of variables in comparison to the number of samples.

## Results

### Uropodina community in badger nests

The analysed nest material from 32 setts included 413 specimens representing 16 species in various developmental stages (adults, protonymphs, and deutonymphs) (Table 1). In total, 31 analysed nests were inhabited by Uropodina mites (there were no Uropodina mites in only one nest). The median number of species per nest was 2.5 (range 0–7). The number of individuals per nest was also variable, with a median of 8.0 (range 0–98). The Uropodina community was dominated by three species: *Trematura patavina* (22.5%, *n* = 93 individuals), *Oodinychus ovalis* (17.2%, *n* = 71), and *Olodiscus minima* (15.5%, *n* = 64) (Table 2). Other nidicolous species occurring in the nests were: *Nenteria oudemansi* (14.8%, *n* = 61), *Phaulodiaspis borealis* (7.0%, *n* = 29), *Phaulodiaspis rackei* (4.6%, *n* = 19), *Uroseius hunzikeri* (1.7%, *n* = 7), *Uropoda orbicularis* (1.5%, *n* = 6), and *Apionoseius infirmus* (1.0%, *n* = 4). The other eurytopic species recorded in this research (e.g., *Olodiscus minima*, *Oodinychus ovalis*, *Oodinychus karawaiewi*) were not associated with nests as their only habitat. The three most frequent species were: *Oodinychus ovalis* (62.5%, *n* = 20 nests), *Nenteria oudemansi* (46.9%, *n* = 15), and *Olodiscus minima* (40.6%, *n* = 13) (Table 2).

**Table 1:** Uropodina species recorded in badger nests and the abundance of adults of both sexes and juvenile stages in 2018–2019

Species	Total	Adults		Juveniles	
		♀♀	♂♂	Deutonymphs	Protonymphs
*Trematura patavina* (Canestrini)	93	13	13	63	4
*Oodinychus ovalis* (C.L. Koch)	71	19	21	25	6
*Olodiscus minima* (Kramer)	64	52	4	8	0
Nenteria oudemansi* (Hirschmann & Z.-Nicol)	61	9	5	44	3
*Phaulodiaspis borealis* (Sellnick)	29	9	6	13	1
*Oodinychus karawaiewi* (Berlese)	27	6	8	11	2
*Phaulodiaspis rackei* (Oudemans)	19	3	7	6	3
*Trachytes aegrota* (C.L. Koch)	14	13	0	1	0
*Uropoda orbicularis* (Müller)	6	0	0	6	0
*Uroseius hunzikeri* (Schweizer)	7	0	0	7	0
*Uropoda* sp.	6	0	0	6	0
*Polyaspinus cylindricus* (Berlese)	5	4	0	1	0
*Apionoseius infirmus* (Berlese)	4	0	0	4	0
*Trachytes pauperior* (Berlese)	4	4	0	0	0
*Urodiaspis tecta* (Kramer)	2	2	0	0	0
*Dinychus arcuatus* (Trägårdh)	1	1	0	0	0
Total	413	135	64	195	19

*First record for Poland

**Table 2:** Dominance and frequency structure of the Uropodina community from badger nests

Dominance class	Species	%	Frequency class	Species	%
D5 eudominants	–		F5 euconstants	*Oodinychus ovalis*	62.5
D4 dominants	*Trematura patavina*	22.5	F4 constants	*Nenteria oudemansi*	46.9
	*Oodinychus ovalis*	17.2		*Olodiscus minima*	40.6
	*Olodiscus minima*	15.5			
D3 subdominants	*Nenteria oudemansi*	14.8	F3 subconstants	*Phaulodiaspis borealis*	28.1
				*Phaulodiaspis rackei*	21.9
				*Trachytes aegrota*	21.9
				*Trematura patavina*	18.8
				*Uropoda orbicularis*	15.6
D2 residents	*Phaulodiaspis borealis*	7.0	F2 accessory species	*Oodinychus karawaiewi*	9.4
	*Oodinychus karawaiewi*	6.5		*Apionoseius infirmus*	9.4
	*Phaulodiaspis rackei*	4.6		*Uroseius hunzikeri*	9.4
	*Trachytes aegrota*	3.4		*Uropoda* sp.	9.4
				*Polyaspinus cylindricus*	6.3
D1 subresidents	*Uroseius hunzikeri*	1.7	F1 accidentals	*Trachytes pauperior*	3.1
	*Uropoda orbicularis*	1.5		*Urodiaspis tecta*	3.1
	*Uropoda* sp.	1.5		*Dinychus arcuatus*	3.1
	*Polyaspinus cylindricus*	1.2			
	*Apionoseius infirmus*	1.0			
	*Trachytes pauperior*	1.0			
	*Urodiaspis tecta*	0.5			
	*Dinychus arcuatus*	0.2			

### Variation in Uropodina community structure

**Fig. 1:**
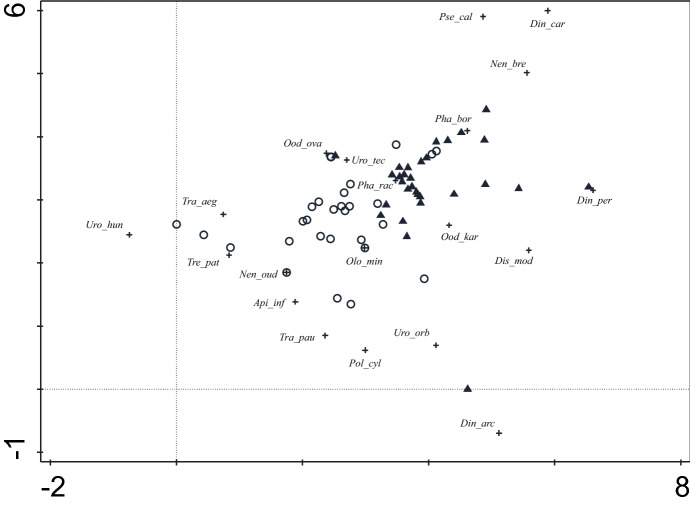
Detrended correspondence analysis (DCA) scatterplot showing the relationships between Uropodina mite communities from 31 badger and 34 mole nests on the first two axes. First and second axis explained 23.6% of variation. Circles = badger nests; triangles = mole nests. Abbreviations of species (marked with +): *Api_inf* = *Apionoseius infirmus*; *Din_arc* = *Dinychus arcuatus*; *Din_car* = *D. carinatus*; *Din_per* = *D. perforatus*; *Dis_mod* = *Discourella modesta*; *Nen_bre* = *Nenteria breviunguiculata*; *Nen_oud* = *N. oudemansi*; *Olo_min* = *Olodiscus minima*; *Ood_kar* = *Oodinychus karawaiewi*; *Ood_ova* = *O. ovalis*; *Pha_bor* = *Phaulodiaspis borealis*; *Pha_rac* = *Ph. rackei*; *Pol_cyl* = *Polyaspinus cylindricus*; *Pse_cal* = *Pseudouropoda calcarata*; *Tra_aeg* = *Trachytes aegrota*; *Tra_pau* = *T. pauperior*; *Tre_pat* = *Trematura patavina*; *Uro_tec* = *Urodiaspis tecta*; *Uro_orb* = *Uropoda orbicularis*; *Uro_hun* = *Uroseius hunzikeri*

In total, 255 individuals of 14 species were recorded in 2019, in comparison to 158 individuals of 13 species in 2018, in spite of the lower number of analysed nests in 2019 (15) than in 2018 (17). There were no significant differences in the number of species per nest between 2018 (median 2, range 0–6) and 2019 (median 3, range 1–7) (*t* = − 0.38, *df* = 30, *p* = 0.70). Also, there were no significant differences in the number of individuals per nest between 2018 (median 7, range 0–26) and 2019 (median 8, range 1–98) (*t* = − 0.95, *df* = 30, *p* = 0.35). The DCA indicated that the Uropodina community from badger nests differed from that of mole nests (Fig. 1). Both groups partially overlap in the middle of the graph, as there are some common nidicolous Uropodina species present in both types of microhabitats (e.g., *O. karawaiewi*, *O. ovalis*, *Ph. rackei*, *Ph. borealis*, *Urodiaspis tecta*). Badger nests were distinguished by two species: *T. patavina* and *N. oudemansi*. These species are on the one end of the marked gradient along the horizontal axis, which may reflect different microhabitat conditions offered by extensive badger setts in comparison to mole burrows. At the other end of the gradient are *Nenteria breviunguiculata*, *Pseudouropoda calcarata*, *Discourella modesta*, *Dinychus carinatus*, and *D. perforates*, which occurred only in mole nests.

## Discussion

A variety of burrow types, differing in internal architecture and size, are dug by birds (Heneberg et al. 2018), moles (Bartkowska 1986), voles (Hackman 1963), and other animals (Celebias et al. 2019). They are known to be rich underground breeding habitats for many groups of arthropods. Despite some publications about their fauna (Payne 1979, 1982; Bartkowska 1986; Hancox 1988; Heneberg et al. 2018), it still needs further explorations because of the great variety of burrow types across regions and also because of the great diversity of arthropod species dwelling in burrows. Recent publications still present new arthropod associations and new aspects of burrow ecology and their importance in ecosystems (Heneberg et al. 2018; Celebias et al. 2019). It is difficult to obtain any data concerning nidicolous fauna from badger setts because of their extensive underground architecture and thus limited access to crucial parts of the setts (chambers with nests). This results in the scarcity of data about any mite species from this specific microhabitat type. Hancox (1988) reviewed data from Europe about arthropods dwelling in badger setts, and found that ca. 89 species of different systematic groups had been reported from them. He listed also some mite species but no Uropodina. The only way to get some relevant data about mites dwelling in badger setts is sampling the nest material excavated by badgers during the cleaning and enlarging of chambers. This is the best opportunity to collect fresh nest material for further analysis of mites. Our preliminary and pioneer research gave us the opportunity to recognize the species diversity of Uropodina mites, with the most complete species list that has ever been published for European badger nests.

All of the 16 Uropodina species presented in this study were never recorded in badger nests before, but some of them were reported from nests of other mammals, such as moles, e.g., *Phaulodiaspis borealis*, *Ph. rackei*, *Uroseius hunzikeri*, *Uropoda orbicularis* (Napierała et al. 2016). The Uropodina community from badger nests differs quantitatively and qualitatively from data obtained for moles (Fig. 1). In some reports, *Phaulodiaspis borealis* and *Ph. rackei* constituted even 60% of the entire community in mole nests (Błoszyk et al. 2003; Napierała and Błoszyk 2013), whereas in this study of badger nests they accounted for < 10%. Another case is represented by *Uroseius hunzikeri*. It is an extremely rare nidicolous species (Napierała and Błoszyk 2013), which was recorded also in this study with a very low abundance and frequency. It seems that *U. hunzikeri* is associated with underground nests in general, rather than with any burrow host species, because it occurs also in mole nests as well as bird burrows of bee-eater (*Merops apiaster*) and sand martin (*Riparia riparia*) (Błoszyk et al. 2006). Other species reported with varying frequency and dominance in badger nests, such as *Apionoseius infirmus* (Błoszyk et al. 2006), were also reported from white-tailed eagle (*Haliaeetus albicilla*) nests, while others from litter or soil: *Olodiscus minima*, *Oodinychus ovalis*, *Oodinychus karawaiewi* (Zduniak et al. 2019). Moreover, we recorded two nidicolous species that reached high abundance in badger nests: *Trematura patavina* and *Nenteria oudemansi.* At the moment, the presence of *Nenteria oudemansi* seems to be characteristic of only badgers because it has never been recorded from nests of other mammals (Fain et al. 1991; Christian 1998; Huhta 2016; Napierała et al. 2016).

The high dominance (> 20% of the community) of *Trematura patavina* in badger setts indicates that its occurrence is not accidental but this kind of microhabitat represents optimal conditions for this species. Few individuals of *T. patavina* have been reported from nests of Middle East blind mole rat *Spalax ehrenbergi* (Hirschmann et al. 1978) and coraciiform birds nesting in tree holes (Fend’a 2009). It was also found under tree bark (Wiśniewski and Hirschmann 1990) and as a phoretic species on *Rhynchophorus ferrugineus* (El-Sharabasy 2010). Wiśniewski and Hirschmann (1990) mentioned that this species was known mostly as only its juvenile stage—protonymphs. In previous studies *T. patavina* was not abundant and only El-Sharabasy (2010) found as many as 272 individuals. In our research this species reached a high abundance and frequency in badger nests. Moreover, we found there all stages of *T. patavina*: protonymphs, deutonymphs, and adults of both sexes (Table 1). This brings new details concerning the biology and ecology of this extremely rare species, suggesting that badger nests are probably its optimal microhabitat, where it can complete its life cycle, which is the first record in case of this mite species.

The second, exclusive dweller of badger nests is *Nenteria oudemansi*, a very rare Uropodina mite species known from few locations across Europe, which was never found in Poland before (Napierała et al. 2009; Błoszyk et al. 2015; Błoszyk and Napierała 2018). It was known from dipper (*Cinclus cinclus*) nests (Fain et al. 1991) but not reported from mammal nests at all (Napierała et al. 2016). Our research revealed that in the Uropodina community of badger nests it was a subdominant (14.8%). Other studies reported its occurrence in decaying hay in Finland (Huhta 2016) and in catacombs in Vienna (Christian 1998), which indicates the preference of *N. oudemansi* for microhabitats like underground nests built of plant material. The high frequency and dominance of this Uropodina mite, with the presence of both sexes and developmental stages in our results, confirms that badger nests are optimal and natural microhabitats for this species.

The high shares of *T. patavina* and *N. oudemansi* in the Uropodina assemblage from badger nests—in comparison to other microhabitats, such as mole nests—may result from the large size and volume of underground corridors and chambers dug by this medium-sized carnivore. Badger setts offer secluded and stable microhabitats that are characterized by specific and constant microclimate due to low temperature fluctuations between day and night all over the year (Moore and Roper 2003). In winter the temperature inside the burrow is usually higher than outside the sett, and in southern England it oscillated around 1.6 °C, with humidity about 85% (Kaneko et al. 2010). The stable microclimate of the burrows is supported by their well-developed underground architecture. Badgers dig tunnels reaching 1.2–1.8 m underground (Fisher and Dunand 2016) and up to 360 m long (Roper 1992). The total volume of underground tunnels and chambers may be reflected in the quantity of excavated soil, ranging between 0.2 and 27.8 m^3^ (Neal and Roper 1991; Coombes and Viles 2015). Beside the well-developed architecture of setts, another important factor supporting convenient conditions for many invertebrate taxa, including Uropodina mites, seems to be that badgers collect a lot of nest material. The quantity of plant material collected in nests and the volume of burrows may create convenient and stable conditions, insensitive to any disturbance, such as drought.

Mole nests are characterised by very diverse and rich Uropodina assemblages (Napierała et al. 2016), to a certain extent similar to the community from badger nests (Fig. 1) because of the occurrence of common nidicolous species, such as *Oodinychus karawaiewi, O. ovalis, Phaulodiaspis rackei*, *Ph. borealis* and *Urodiaspis tecta*. However, our results revealed that—in contrast to moles—the Uropodina community of badger nests is distinguished by a high abundance of *Trematura patavina* and the first record of *Nenteria oudemansi* from underground mammal nests. In addition to previous studies of the impact of badgers on local fauna and flora (Sleeman et al. 1995, 1997; Kurek and Cykowska-Marzencka 2016; Rola et al. 2017), the results presented here indicate that the European badger setts affect both aboveground and underground biodiversity.
